# Changes in standing stability with balance‐based torso‐weighting with cerebellar ataxia: A pilot study

**DOI:** 10.1002/pri.1814

**Published:** 2019-11-20

**Authors:** Gail L. Widener, Nicole Conley, Sarah Whiteford, Jason Gee, Anthony Harrell, Cynthia Gibson‐Horn, Valerie Block, Diane D. Allen

**Affiliations:** ^1^ Department of Physical Therapy Samuel Merritt University Oakland CA USA; ^2^ Graduate Program in Physical Therapy University of California San Francisco/San Francisco State University San Francisco CA USA; ^3^ Department of Neurology University of California San Francisco San Francisco CA USA

**Keywords:** cerebellar disorders, motor ataxia, postural balance, rehabilitation

## Abstract

**Objectives:**

People with cerebellar ataxia have few options to improve the standing stability they need for function. Strategic placement of light weights on the torso using the balance‐based torso‐weighting (BBTW) method has improved stability and reduced falls in people with multiple sclerosis, but has not been tested in cerebellar ataxia. We examined whether torso‐weighting increased standing stability and/or functional movement in people with cerebellar ataxia.

**Methods:**

Ten people with cerebellar ataxia and 10 matched controls participated in this single‐session quasi‐experimental pilot study. People with ataxia performed the Scale for the Assessment and Rating of Ataxia (SARA) prior to clinical testing. All participants donned inertial sensors that recorded postural sway; stopwatches recorded duration for standing and mobility tasks. All participants stood for up to 30 s on firm and foam surfaces with eyes open then eyes closed, and performed the Timed Up and Go (TUG) test. Light weights (0.57–1.25 kg) were strategically applied to a vest‐like garment. Paired *t* tests compared within‐group differences with and without BBTW weights. Independent *t* tests assessed differences from controls. All *t* tests were one‐tailed with alpha set at .05.

**Results:**

Duration of standing for people with ataxia was significantly longer with weighting (*p* = .004); all controls stood for the maximum time of 120 s with and without weights. More severe ataxia according to SARA was moderately correlated with greater improvement in standing duration with BBTW (Pearson *r* = .54). Tasks with more sensory challenges (eyes closed, standing on firm surface) showed less body sway with weighting. Duration for the TUG was unchanged by torso‐weighting in people with ataxia.

**Conclusion:**

Strategic weighting improved standing stability but not movement speed in people with ataxia. BBTW has potential for improving stability and response to challenging sensory conditions in this population. Future studies should further examine gait stability measures along with movement speed.

## 1 INTRODUCTION

In people with cerebellar ataxia (CA), dyscoordinated movement can result in postural instability, falls, and associated injuries (Rüb et al., 2008). Controlled movement depends on a person's ability to perceive self‐motion and coordinate muscle activity within and between body parts to perform the desired task efficiently (Marsden, 2018). The normally functioning cerebellum plays a key role in motor control by comparing an efferent copy of the motor command with sensory information resulting from truncal, limb, and ocular movement (Ioffe, Chernikova, & Ustinova, 2007; Marsden, 2018). When the cerebellum degenerates, as in CA, damaged neurons and their connections with the brainstem areas, contralateral motor cortex, or vestibular nuclei can impair the cerebellum's ability to integrate sensory input sufficiently to monitor and correct motor commands and their consequences (Marsden, 2018). The result can include increases in postural sway, slowed or hypermetric responses to balance perturbations, and irregular stepping during gait (Marsden, 2018; Stephen et al., 2019). Few treatment options have been identified that address instability and fall risk in this population. No drugs or medications have been approved by the U. S. Food and Drug Administration for the treatment of CA or the motor features associated with ataxia (Ilg et al., 2014; Sarva & Shanker, 2014; Stephen et al., 2019). Evidence of the effectiveness of rehabilitation is not conclusive, with partial response to physical therapy for some patients (Fonteyn et al., 2014; Milne, Corben, Georgiou‐Karistianis, Delatycki, & Yiu, 2017). Researchers state that evidence‐based guidelines for the rehabilitation of people with CA need to be developed (Ilg et al., 2014). The purpose of this pilot study was to determine if a single session of strategic torso‐weighting (Gibson‐Horn, 2008) changed immediate standing stability and/or functional movement in people with CA.

Postural instability in individuals with ataxia may manifest as increased variability of amplitude and direction of movement, particularly at the trunk, (Buckley, Mazzà, & McNeill, 2018; Conte et al., 2014; Martino et al., 2014) with associated imbalance, unsteadiness, and inconsistent gait patterns (Stolze et al., 2002; Wuehr, Schniepp, Ilmberger, Brandt, & Jahn, 2013). Imbalance can increase the risk of falling for people with ataxia (Fonteyn et al., 2013; Van de Warrenburg, Steijns, Munneke, Kremer, & Bloem, 2005). Falls can generate apprehension and lead to decreases in functional independence and physical activity (Schniepp et al., 2014).

Research in cerebellar ataxia supports commonly used rehabilitative techniques such as the weighting of trunk, extremities, or devices (Clopton, Schultz, Boren, Porter, & Brillhart, 2003; Marsden, 2018; Morgan, 1975; Stephen et al., 2019), whereas other research describes benefits from balance and coordination exercises (Ilg et al., 2010; Miyai et al., 2012), visual and auditory feedback during gait, and body‐weight‐supported treadmill walking (Balliet, Harbst, Kim, & Stewart, 1987; Marquer, Barbieri, & Pérennou, 2014). No studies have specifically examined balance‐based torso‐weighting (BBTW; Gibson‐Horn, 2008) in people with CA, although it has resulted in improvements for people with multiple sclerosis (MS) in static standing, gait velocity, cadence, and percent of gait cycle in single‐limb support (Crittendon, O'Neill, Widener, & Allen, 2014; Gorgas, Widener, Gibson‐Horn, & Allen, 2015). Because up to 80% of people with MS experience ataxia, (Mills, Yap, & Young, 2007), people with CA may also benefit from BBTW.

BBTW involves assessment of a standing individual's directional instability while a trained clinician manually applies perturbations and resisted rotations at the shoulders and pelvis. The clinician strategically places light weights (objects with designated mass) on the trunk to counter instability. The mechanism for the effectiveness of BBTW is not specifically known. Prior work has disproven a strictly biomechanical mechanism (Crittendon et al., 2014) and argued against alternative hypotheses such as joint compression or increased conscious awareness proposed when heavier weights are used (Hunt, Widener, & Allen, 2014). The mechanism likely involves enhanced sensory input to improve perception of self‐motion and position (Crittendon et al., 2014). The theoretical rationale for proposing BBTW for people with CA is that a stronger sensory signal may help compensate for sensory integration deficits resulting in ataxia and postural control issues in this population.

Our study focused on scientific feasibility according to Thabane et al.'s (2010) recommendations for initial pilot testing (Thabane et al., 2010), examining the possibility of response to the intervention in people with CA. We hypothesized that participants would show improvement in standing stability and/or functional movement compared with a no‐weight condition. We also tested age‐ and sex‐matched controls to validate management feasibility, that is, the ability of the protocol to result in usable data (Thabane et al., 2010).

## 2 METHODS

This was a quasi‐experimental pilot study examining postural and mobility responses with and without BBTW in a single session. This study was approved by the appropriate university ethics committees. All participants provided written informed consent.

Participants included people with CA and age‐ and sex‐matched controls. Inclusion criteria for all participants were

1. English‐speaking,
2. age 18 years or older,
3. ambulatory for at least 20 ft. with a cane or no assistive device, and
4. able to stand unsupported for 60 s

Further, participants in the CA group must have

- diagnosis of CA with dominant‐inherited, recessively inherited or sporadic ataxia and
- self‐reported balance or mobility difficulties.

Participants were excluded from the study if they were

1. unable to comprehend and follow instructions,
2. diagnosed with a concurrent neurological disorder such as MS, head injury, or stroke,
3. had pain that could be exacerbated by external perturbation while standing,
4. had resting blood pressure outside the pre‐set limit (>150/90 mm Hg), or
5. could not lift their toes off the floor or lift thighs (one at a time) off the chair when sitting (such weakness may restrict a person's ability to respond to BBTW based on previous clinical observations).

Participants with CA were recruited from attendees of the National Ataxia Foundation Annual Meeting in Las Vegas in 2014. The number in this sample of convenience was limited by time available to test participants in single sessions (about 2 hr each) in a separate room during the meeting. Sex‐ and age‐matched controls were recruited 6 months later through flyers and online postings and tested at Samuel Merritt University.

Using standardized terminology and recommendations for pilot studies (Thabane et al., 2010), we focused on scientific and management feasibility of BBTW in people with CA. Scientific feasibility was determined by a significant response of participants to weighting. Clinical testing was performed with no weight first (in prior studies, some participants maintained stability changes for over an hour after weights were removed). Management feasibility (Thabane et al., 2010) examined measures new to this research (inertial sensors to assess body sway) by testing age‐ and sex‐matched controls and confirming differences in performance between those with and without movement disorders observed in prior studies (Crittendon et al., 2014; Gorgas et al., 2015).

The outcome measures used in this study were the modified Clinical Test of Sensory Interaction on Balance (mCTSIB; Cohen, Blatchly, & Gombash, 1993; Wrisley & Whitney, 2004) and the Timed Up and Go (TUG; Podsiadlo & Richardson, 1991) We chose these measures to test participants' performance in both static and dynamic balance tasks. For standing stability (mCTSIB), participants stood on firm and foam surfaces, with eyes open and eyes closed for 30 s each (or for shorter time if the person moved their feet out of position or needed assistance to prevent falling). Each of the four standing stability tasks was performed once. For dynamic stability (TUG test), participants stood up from a chair, walked 3 m, turned around, walked back, and sat down in the chair again (Podsiadlo & Richardson, 1991). The TUG was performed twice. The first trial was for practice, and the second trial was recorded. Rest breaks were provided as needed between trials. The participants performed all testing tasks without weights before undergoing the BBTW procedure. After receiving BBTW weights, participants repeated the standing stability and TUG tasks.

### 2.1 BBTW procedure

To control for possible inter‐rater differences, one physical therapist (CGH) with over 30 years of clinical experience in balance and gait rehabilitation performed all BBTW procedures for this study. The therapist started balance assessment by observing the relative amount and direction of sway while the participant stood quietly (Gibson‐Horn, 2008). The therapist then applied anterior, posterior, and lateral perturbations (nudges) at the shoulders and pelvis to observe the participant's response and direction of balance loss. Rotational forces were applied manually through the shoulders and pelvis to determine asymmetries in the participant's ability to maximally resist while maintaining upright balance. In prior studies, this therapist used a handheld dynamometer to standardize and confirm the reliability of perturbations and rotational forces applied during the BBTW procedure (Crittendon et al., 2014). Loss of balance during perturbations and rotational forces was scored on a 0–4 scale developed to facilitate application of weights in the BBTW procedure (Allen et al., 2018). Responses were scored: (0) No balance loss, brisk response to perturbation; (1) Minimal balance loss, delayed onset of return to upright, (2) Moderate balance loss, large trunk movement or parachute reaction with no foot movement; (3) Moderate–severe balance loss, large trunk movement with foot movement or takes a small step; (4) Severe balance loss, manual contact by the researcher required to prevent a fall. The scale has shown good to excellent inter‐rater agreement (Allen et al., 2018) indicating that other therapists can observe balance loss similarly.

Weights (0.06, 0.11, or 0.23 kg) were placed using Velcro on a size‐adjustable vest‐like garment (BalanceWear, Motion Therapeutics, Oxnard, CA). Weight location was customized to counter the individual's direction of balance loss, asymmetry of resistance, and latency of response to perturbations. For example, if a participant showed a 3 on the balance loss scale when attempting to resist pelvic rotation to the right, a 0.23 kg weight affixed to the left low back region would assist the participant to maintain balance and resist this force. Balance was reassessed with weights in place to confirm that greater stability and/or quicker response were demonstrated. Better response was indicated by reduction of balance loss scores to 0 or 1 with reassessment. Weight amount and location were recorded. In addition, we recorded participants' tolerance of the intervention and any adverse events that occurred.

### 2.2 Data analysis

Primary outcomes for standing stability were “duration of standing” recorded by stopwatch (starting once participants were in position and ready) and “body sway” as recorded by body‐worn inertial sensors (APDM Wearable Technologies, Portland, OR), technology used effectively in other populations (Deshmukh, Russell, Lucarino, & Robinovitch, 2012; Spain et al., 2012). According to manufacturer suggestions, the sensors were placed at the lumbar spine, anterior sternum, bilateral ankles, and bilateral wrists (Horak, King, & Mancini, 2015), and recorded movement with an accelerometer, gyroscope, and magnetometer. Software combined information from individual sensors to record body sway as 95% of the ellipse sway area, defined as “the area of an ellipse covering 95% of the sway angle in both the coronal and sagittal planes” (Whitepaper, 2015). A lower number for sway area indicates greater stability.

The primary outcome for the TUG was time. The participant was seated with back touching the chairback: Time started when the participant was told to “go”; time stopped when the participant's back touched the chairback again after walking. Secondary TUG outcomes included turn duration, sit to stand duration, and peak turn velocity recorded by the inertial sensors (Salarian et al., 2010).

In addition to the primary outcome measures, participants completed the Activities‐specific Balance Confidence (ABC) scale (Myers, Fletcher, Myers, & Sherk, 1998). A score of 80% or more suggests a high level of physical function or balance confidence (Myers et al., 1998). A score of less than 67% in elderly individuals is predictive of a future fall (Lajoie & Gallagher, 2004). All participants self‐reported the number of falls they had experienced in the previous 6 months. Participants with ataxia also underwent testing using the Scale for the Assessment and Rating of Ataxia (SARA; Schmitz‐Hubsch et al., 2006; Schmitz‐Hubsch et al., 2010) to provide an indication of ataxia severity. The SARA records performance in eight different tasks including balance, gait, and movements of the upper and lower extremities. The SARA scale has a 0–40 range with higher scores representing higher levels of ataxia‐related disability.

To describe the sample, we recorded mean and standard deviation for age, ABC scale, and performance on the SARA. We calculated associations between measures using Pearson *r* correlations. To compare performance results, we used paired *t* tests within groups (no weight compared with BBTW weighted condition) and independent *t* tests between groups. Alpha was set at .05, using one‐tailed tests because the research hypotheses were unidirectional. Comparisons and correlations were performed using Excel 2013; effect sizes were calculated using equations for effect size *d* for unpaired and paired *t* tests (Portney & Watkins, 2009) and comprehensive meta‐analysis software (Borenstein, Hedges, Higgins, & Rothstein, 2005).

## 3 RESULTS

Eleven people with ataxia participated. One participant (A05) did not meet resting blood pressure criteria, and further testing for this participant was discontinued. Included participants (Table 1: 6 female, 4 male) had mean (SD [standard deviation]) age of 47.2 years (6.6), SARA score of 12.5 (4.16), and ABC score of 54.6 (16.8). The correlation (*r*) between age and ABC score was −0.75: Younger people rated themselves as more confident. The correlation between age and total SARA scores was −0.23: Age did not significantly correlate with dyscoordinated movement. In the previous 6 months, participants with ataxia reported a mean (SD) of 15.1 (27.4) falls per person, a number that did not correlate highly with age (*r* = .18). For the weighting condition, participants were weighted with a total ranging from 0.68 to 1.23 kg (equivalent to 0.7% to 1.4% of body mass).

**Table 1:** Participants with ataxia

Participant	Age	Type of ataxia	Year diagnosed with ataxia	ABC score	SARA	Falls in the past 6 months
AO1	52	Unknown	2011	56.9	9	1
AO2	49	SCA	1998	44.4	8.5	0
AO3	39	SCA 2	2003	84.4	17.5	2
AO4	51	ARCA‐1	1998	51.3	14.5	25
AO6	51	SCA (sporadic)	2006	42.5	15	90
AO7	35	Unknown	1996	78.8	13.5	10
AO8	50	SCA 8	2004	28.1	13.5	6
AO9	45	SCA 2	Unknown	60.6	18	1
A10	57	Unknown	2008, confirmed 2014	46.9	10.5	4
A11	43	SCA 3	2014	51.9	5	12
**Average (SD)**	47.2 (6.6)			54.6 (16.8)	12.5 (4.2)	15.1 (27.4)

Abbreviations: ABC, Activities‐specific Balance Confidence; ARCA, autosomal recessive cerebellar ataxia; SARA, Scale for the Assessment and Rating of Ataxia; SCA, spinocerebellar ataxia; SD, standard deviation.

Ten gender‐ and age‐matched control participants were recruited. The mean (SD) age was 47.8 (8.8), and ABC score was 97.5 (1.54). Participants without ataxia had significantly higher ABC scores when compared with participants with ataxia (*p* < .001). In the previous 6 months, controls reported a mean (SD) of 0.33 (0.7) falls per person. For the weighting condition, controls were weighted with a total ranging from 0.11 to.86 kg (equivalent to.1% to 1% of body mass). All participants in both groups tolerated the weighting intervention with no adverse events.

### 3.1 Standing stability

**Figure 1:**
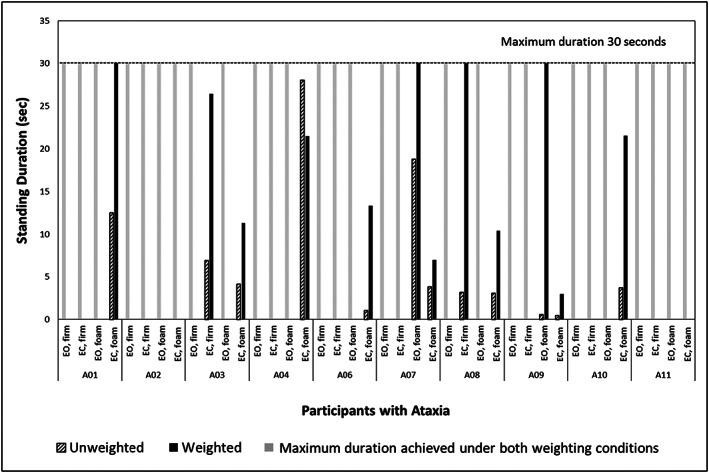
Amount of time participants with ataxia spent in each standing task before needing assistance (to a maximum of 30 seconds). All healthy controls reached the maximum time of 30 s for all tasks while unweighted and weighted. EO, eyes open; EC, eyes closed

All control participants stood for the full 30 s during each task and condition with and without weighting (ceiling effect). Total stand time for persons with ataxia without weights averaged 23.15 s (5.6) per standing trial (between group *p* = .002, effect size *d* = −1.7, 95% CI [−2.72, −0.68]). Total stand time for participants with ataxia with BBTW weights was significantly longer, averaging 26.86 s (2.6) per activity (within group *p* = .004, effect size *d* = 2.0, 95% CI [0.93, 3.07]). In a post hoc analysis of standing duration (Figure 1), standing trials were categorized by the number of sensory modalities constrained:

- Eyes open on firm surface had “no” sensory modalities constrained: All participants with ataxia stood for the full 30 s without and with weights;
- Eyes open on foam surface (affecting the somatosensory system) and eyes closed on firm surface (affecting the visual system) each had “one” modality constrained: Participants with ataxia increased standing duration with weights, within group *p* = .03;
- Eyes closed on foam surface constrained “two” modalities, including both the somatosensory and visual systems: Participants with ataxia improved with weights, within group *p* = .02.

**Figure 2:**
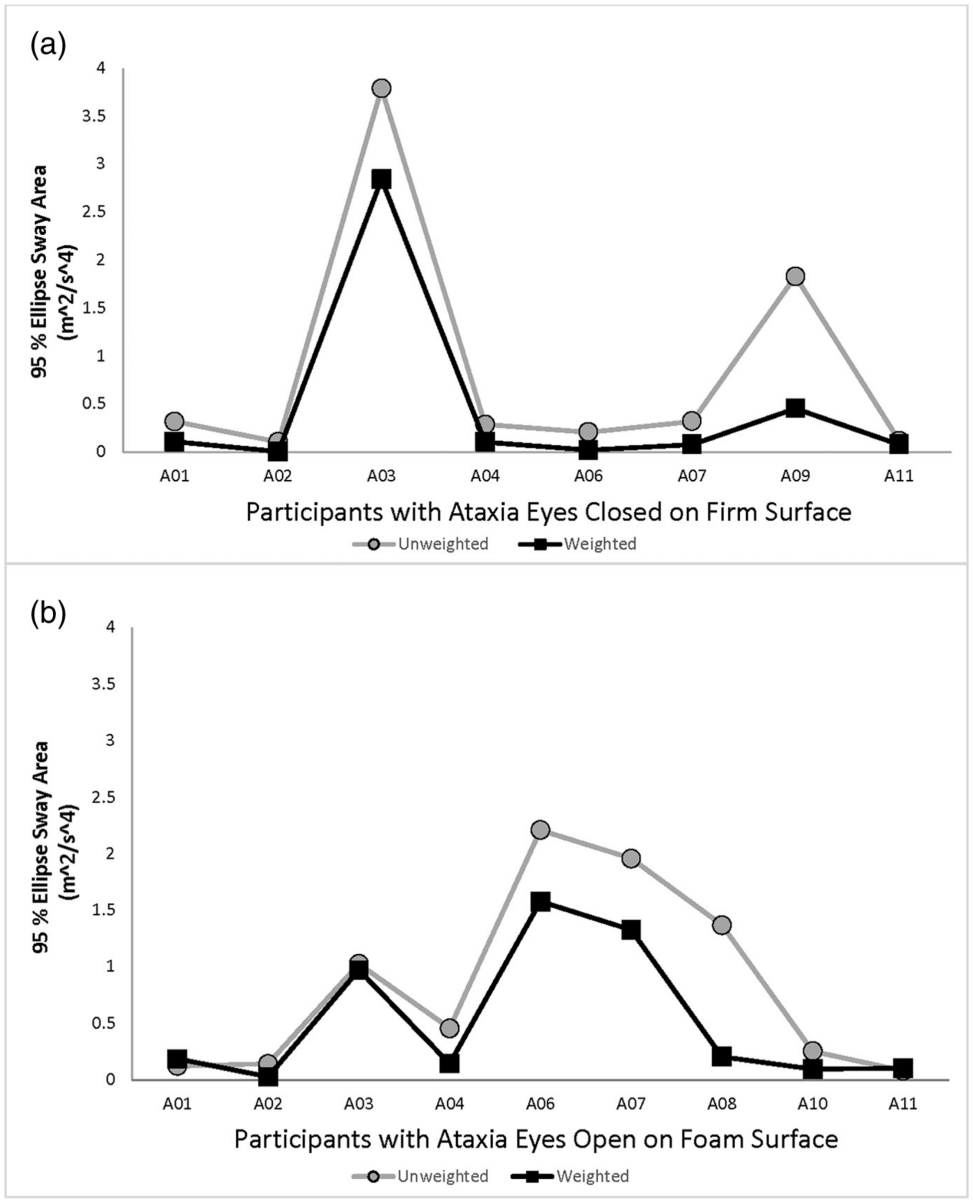
95% Ellipse away area for participants with ataxia during a) eyes closed on firm surface, *n* = 8, within‐group differences in unweighted and BBTW conditions, *p* = .020; and b) eyes open on foam, *n* = 9, within‐group differences in unweighted and BBTW conditions, *p* = .02. Sway area generally decreased with torso weighting

Controls showed no significant difference in sway area between no weight and BBTW weighted conditions for any standing tasks (Table 2). The sway area during no‐weight standing tasks was significantly less for controls than for people with ataxia (between group *p* = .006, effect size *d* = 1.39, 95% CI [0.41, 2.36]). People with ataxia showed within‐group sway area differences with BBTW compared with no weights for some standing tasks (Figure 2a,b) but not others:

- eyes open, standing on foam surfaces, yes (*p* = .02; effect size *d* = 1.2, 95% CI [0.2, 2.2]);
- eyes closed, standing on firm surfaces, yes (*p* = .02; effect size *d* = 1.4, 95% CI [0.31, 2.49]);
- eyes open on firm surfaces, no (*p* = .23)
- eyes closed on foam surfaces, no (*p* = .48).

Technical difficulties with recordings from the inertial sensors prohibited retrieval of sway area data for some trials for the participants with ataxia: eyes open on firm surface (A01), eyes open on foam (A08 and A10), eyes closed on firm surface (A01), and eyes closed on foam (A09).

**Table 2:** Results of postural sway in standing and TUG tests

	Participants with ataxia (*n* = 8* or 9)			Controls (*n* = 10)		
	No weights mean (SD)	Weights mean (SD)	*p* value†	No weights mean (SD)	Weights mean (SD)	*p* value†
95% Ellipse sway area (m^2^/s^4^)						
EO on firm	0.10 (0.12)	0.06 (0.10)	.23	0.001 (0.0004)	0.001 (0.0004)	.08
EO on foam	0.85 (0.83)	0.52 (0.60)	.02	0.005 (0.003)	0.01 (0.002)	.48
EC on firm*	0.87 (1.31)	0.46 (0.97)	.02	0.001 (0.0006)	0.002 (0.003)	.12
EC on foam	11.24 (10.69)	11.02 (10.12)	.48	0.02 (0.008)	0.02 (0.01)	.38
Durations in seconds; peak turn velocity in deg/s						
TUG	13.98 (4.32)	13.89 (4.38)	.43	6.30 (0.50)	5.99 (0.54)	<.001
TUG turn	3.52 (1.33)	3.38 (0.88)	.27	1.87 (0.30)	1.75 (0.44)	.14
Sit/stand	2.90 (1.42)	2.68 (0.63)	.33	2.19 (0.22)	2.27 (0.27)	0.47
Peak turn velocity	138.35 (42.89)	136.39 (52.13)	.42	250.57 (59.48)	255.34 (87.59)	0.32

† within group, one‐tailed, paired *t* test

Abbreviations: EC, eyes closed; EO, eyes open; SD, standard deviation; TUG, Timed Up and Go.

For participants with ataxia, SARA scores correlated with standing stability measures in the no‐weight condition: total standing duration, *r* = −.7; sway area eyes open on foam surface, *r* = .45; and sway area eyes closed on firm surface, *r* = .7. SARA scores also moderately correlated with change in total standing duration (weighting time minus no‐weighting time) with *r* = .54. Age did not correlate with change in standing duration (*r* = −.11) in our study.

### 3.2 Timed Up and Go

**Figure 3:**
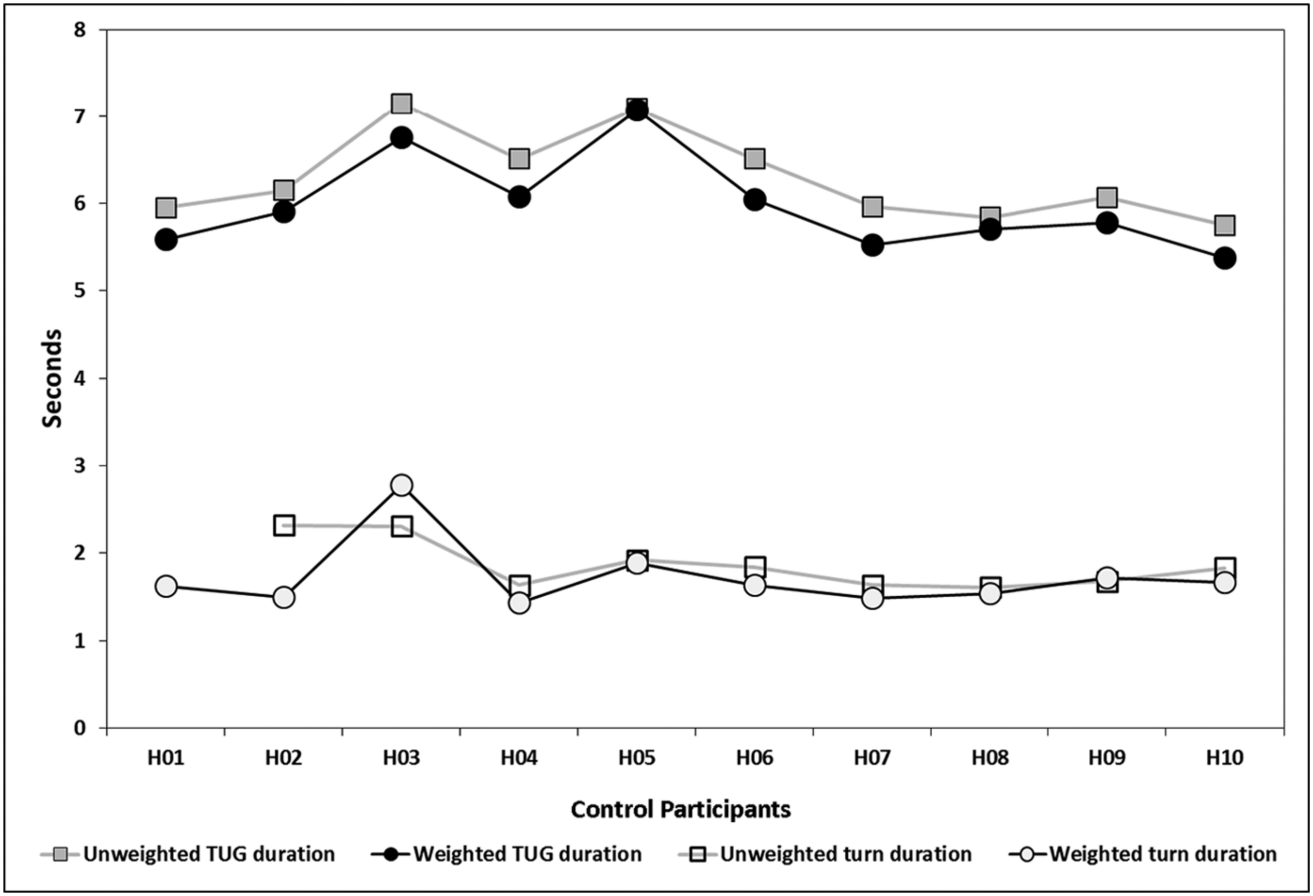
Total TUG duration and turn duration, unweighted versus weighted trials in controls (participants without ataxia). TUG, Timed Up and Go

**Figure 4:**
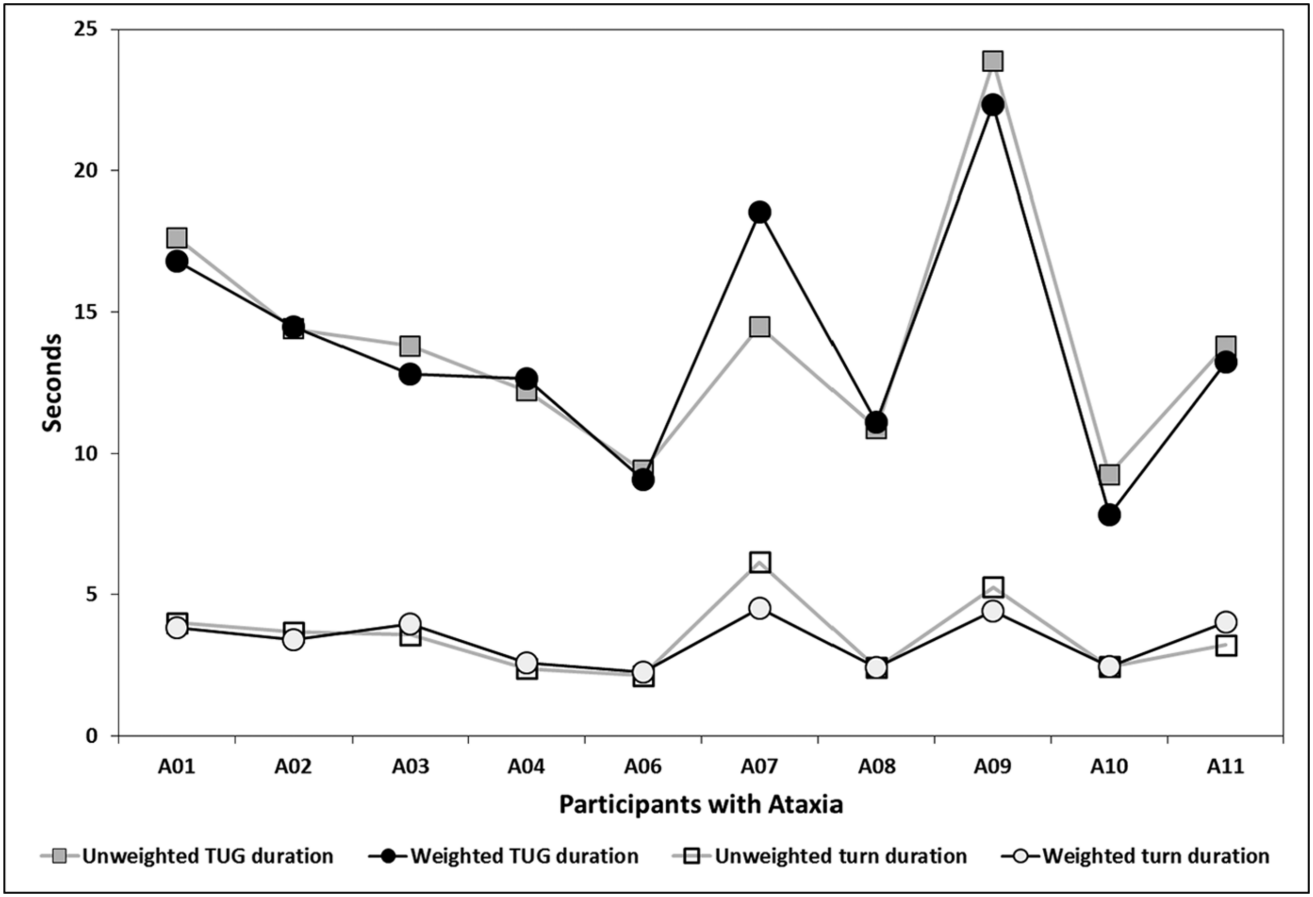
Total TUG duration and turn duration, unweighted versus weighted trials in participants with ataxia. TUG, Timed Up and Go

In controls, time decreased from no weight to BBTW weighted trials in overall TUG duration (within group *p* < .001, Figure 3, effect size *d* = −3.1, 95% CI [−4.4, −1.8]), but not in turn duration (*p* = .14, Figure 3), sit to stand duration (*p* = .47), or peak turn velocity (*p* = .32; Table 2). Participants with ataxia had significantly longer TUG durations compared with the controls (between group *p* < .001; effect size *d* = 2.5, 95% CI [1.33, 3.67]). Participants with ataxia did not change with weighting in TUG duration (within group *p* = .43, Figure 4), turn duration (*p* = .27, Figure 4), sit to stand duration (*p* = .33), or peak turn velocity (*p* = .42). Technical difficulties with inertial sensors prohibited retrieval of TUG turn and sit to stand data for control participant H01.

## 4 DISCUSSION

Ten participants with ataxia and 10 age‐ and sex‐matched controls participated in this study. All but two participants with ataxia had ABC scores below 67% indicating increased fall risk (Lajoie & Gallagher, 2004), whereas controls averaged 97% indicating high balance confidence. Reported falls experienced in the last 6 months also differed: averaging .33 per person for controls versus 15.1 for those with ataxia. Participants with ataxia scored an average of 12.5 on the SARA indicating they had mild to moderate levels of ataxia.

People with ataxia showed within‐group standing stability improvements with BBTW, as hypothesized. However, TUG duration did not change when this cohort wore BBTW weights. The measures used in this study confirmed between‐group differences: Controls showed better standing stability and TUG times compared with people with ataxia. Also, all controls scored above 80% on the ABC indicating a low risk of falls (Myers et al., 1998).

Improvements in standing duration varied based on the number of sensory constraints provided in different standing tasks. A ceiling effect was noted when all sensory modalities were available for balance (eyes open on firm surface): All participants in both groups had a maximum score of 30 s (see Figure 1). With one modality constrained (eyes closed on firm or eyes open on foam surfaces), participants with ataxia were able to stand for up to 30 s with BBTW. For example, participants A07 and A09 could not adequately use vision (eyes open) to compensate for the somatosensory challenge of standing on a compliant (foam) surface without BBTW, but could stand for the full 30 s with BBTW (Figure 1). With two modalities constrained (eyes closed on foam surface), participants with ataxia also improved with BBTW, but standing duration did not reach 30 s. BBTW during this single session seems to suffice as compensation for a single‐modality constraint (vision or somatosensation), but was insufficient to completely compensate when two sensory modalities were constrained in this small sample.

Improvements in sway area for people with ataxia also varied based on sensory constraints provided in different standing tasks (see Table 2). Weighting resulted in a significantly smaller sway area when one sensory modality was constrained (*p* = .03) although the resultant sway area did not match that of controls. Participants with ataxia showed no improvements with zero constraints (eyes open on a firm surface) or two constraints (eyes closed on a foam surface). The magnitude of the sway area was small with both no weights and weights when participants stood with zero constraints: A ceiling effect likely limited change. Sway area was 100 times larger when participants with ataxia stood with two constraints. BBTW did not show an average improvement in the two‐constraint case, possibly because the compensatory weight was not sufficient, the responses were too highly variable, or additional sessions and practice are needed to show an average improvement when the sway area is so markedly enlarged.

The moderate correlation between SARA score and change in standing duration implies that people with more ataxia tend to change more with BBTW than people with less ataxia. A caveat to this interpretation is that immediate weighting effects did not completely compensate for instability when two sensory systems were constrained.

People with ataxia did not move faster during the TUG when weighted. These data contrast with previous studies showing that BBTW decreased TUG duration for people with MS (Widener, Allen, & Gibson‐Horn, 2009). Perhaps this disparity reflects differences in locations of pathology and pathological processes of these two diseases. Not all people with MS have cerebellar lesions; further, the classic demyelination in MS coincides with slowed neural conduction that is not necessarily present in CA. We hypothesize that the increased sensory input provided by BBTW may have improved the coordination but not the speed of movement. Some authors have suggested that decreased gait speed may in itself be a compensatory strategy for the high variability of stepping in CA (Marsden, 2018). Other researchers applying proprioceptive input have noted changes in postural control and stepping variables in people with CA but no significant changes in gait speed (Leonardi et al., 2017). Despite lack of speed improvements, other changes may have occurred for our participants: One participant with ataxia with slower TUG performance volunteered that with BBTW, “It was much easier to stand up from the chair.” Further research investigating the kinetic and kinematics of movement may reveal additional changes in coordination while weighted.

Unlike the participants with ataxia, 10 out of 10 controls decreased TUG duration with weighting, likely a reflection of increased straight‐course gait speed because duration of turns and transfers did not change. These results correspond to improved gait velocity with BBTW in people with MS noted in a previous study (Gorgas et al., 2015). Controls did not change standing stability with weighting because pretest values were already at the ceiling of these measures.

### 4.1 Study limitations

No minimal clinically important differences or minimal detectable changes have been established for mCTSIB standing duration, postural sway, or the TUG in people with CA. However, all effect sizes for significant variables in this study were large (*d* was greater than 0.8), and the confidence intervals did not cross zero. Future studies are needed to determine if long‐term use of BBTW results in benefits in the daily lives of patients with CA.

The sample sizes were small and technical difficulties with the inertial sensors restricted sample sizes further. In addition, large standard deviations indicating great amounts of variability among participants also contributed to large confidence intervals. Future studies with larger samples will likely increase the precision of the estimated effect sizes. Despite these challenges, the power was 79% to over 90% for the measures that were significant in this study. Modifications to the protocol to ensure proper functioning of the equipment (e.g., inertial sensors) are recommended prior to determining sample size needed for a larger study.

In our single‐session design, we had no data regarding possible effects of BBTW on fall incidence. Future studies with longer term use of BBTW could prospectively record differences in fall rate. Blinding of assessors could help minimize possible bias. Generalization of these results to clinical use should take into account that using and applying the BBTW method requires a training course consisting of 2 days in person and 2‐4 hours on‐line instruction.

### 4.2 Clinical implications and future research

The results from this study provide evidence of the scientific feasibility of BBTW in this population. Sway magnitude for people with ataxia was considerably more than the sway of controls even when weighted. We contend that reduction in sway was clinically meaningful because participants with ataxia were able to stand statically for longer durations with BBTW (*p* = .004). One specific concern in CA is the potential for a worse ataxia or tremor when the weights are removed (Marsden, 2018). No signs of worsening or other adverse events were noted when these light weights were removed in this study; clinical experience has shown that other populations typically retain some benefit from BBTW for minutes to hours following removal.

Evidence of management feasibility was mixed. Technical difficulties with recording from the inertial sensors hindered extraction of usable data especially during the performance of the TUG. However, these measures might show improved management feasibility with appropriate protocol modifications. For example, in order for the inertial sensors to collect gait velocity during the TUG, the walk distance must be extended. However, despite the technical difficulties, the controls responded to BBTW as in prior studies, (Crittendon et al., 2014; Gorgas et al., 2015) supporting management feasibility of the protocol. Despite hindrances, this study validated hypothesized between‐group differences.

Because standing stability improved with weighting but movement speed did not in people with ataxia, additional variables should be considered for greater scientific feasibility. People with ataxia may use slower movement as a coping strategy to accomplish tasks (Bastian, 1997), with the speed–accuracy trade‐off formalized by Fitts' law (Fitts, 1954). Testing accuracy (such as step length, step width, and percent of gait cycle in single‐ and double‐limb support during gait) could supplement tests of speed. Further, additional practice time with BBTW applied for long‐term use may allow for participants to adjust strategies and may better reflect the effect BBTW has on both speed and accuracy in people with ataxia.

## 5 CONCLUSIONS

This pilot study provides evidence of scientific feasibility of BBTW for improving standing stability in participants with ataxia. BBTW may be particularly useful in increasing stability in this population when one sensory modality is constrained. Between‐group differences provide evidence of management feasibility. Modifications for future research in people with ataxia might examine measures of accuracy along with speed of functional movement.

## CONFLICT OF INTEREST

The authors declare no conflict of interest.

## References

[pri1814-bib-0001] Allen, D. D. , Magdalin, C. , Schultz, A. , Scott, K. , Jang, C. , Hughes, R. , … Widener, G. L. (2018). Interrater reliability of the balance‐based torso‐weighting method of altering balance and gait [abstract RH20]. International Journal of MS Care, 20(Supplement I), 113.

[pri1814-bib-0002] Balliet, R. , Harbst, K. B. , Kim, D. , & Stewart, R. V. (1987). Retraining of functional gait through the reduction of upper extremity weight‐bearing in chronic cerebellar ataxia. International Rehabilitation Medicine, 8, 148–153. 3610487 10.3109/03790798709166204

[pri1814-bib-0003] Bastian, A. J. (1997). Mechanisms of ataxia. Physical Therapy, 77, 672–675. 10.1093/ptj/77.6.672 9184691

[pri1814-bib-0004] Borenstein, M. , Hedges, L. , Higgins, J. , & Rothstein, H. (2005). Comprehensive meta‐analysis (version 2). Englewood, NJ: Biostat.

[pri1814-bib-0005] Buckley, E. , Mazzà, C. , & McNeill, A. (2018). A systematic review of the gait characteristics associated with cerebellar ataxia. Gait & Posture, 60, 154–163. 10.1016/j.gaitpost.2017.11.024 29220753

[pri1814-bib-0006] Clopton, N. , Schultz, D. , Boren, C. , Porter, J. , & Brillhart, T. (2003). Effects of axial loading on gait for subjects with cerebellar ataxia: preliminary findings. Neurology Report, 27, 15–21. 10.1097/01253086-200327010-00004

[pri1814-bib-0007] Cohen, H. , Blatchly, C. A. , & Gombash, L. L. (1993). A study of the clinical test of sensory interaction and balance. Physical Therapy, 73, 346–351. 10.1093/ptj/73.6.346 8497509

[pri1814-bib-0008] Conte, C. , Pierelli, F. , Casali, C. , Ranavolo, A. , Draicchio, F. , Martino, G. , … Serrao, M. (2014). Upper body kinematics in patients with cerebellar ataxia. Cerebellum, 13, 689–697. 10.1007/s12311-014-0586-z 25063003

[pri1814-bib-0009] Crittendon, A. , O'Neill, D. , Widener, G. L. , & Allen, D. D. (2014). Standing data disproves biomechanical mechanism for balance‐based torso‐weighting. Archives of Physical Medicine & Rehabilitation, 95, 43–49. 10.1016/j.apmr.2013.08.235 24001445 PMC3918424

[pri1814-bib-0010] Deshmukh, P. M. , Russell, C. M. , Lucarino, L. E. , & Robinovitch, S. N. (2012). Enhancing clinical measures of postural stability with wearable sensors. Conference Proceedings IEEE Engineering and Medical Biology Society, 2012, 4521–4524. 10.1109/EMBC.2012.6346972 23366933

[pri1814-bib-0011] Fitts, P. M. (1954). The information capacity of the human motor system in controlling the amplitude of movement. Journal of Experimental Psychology, 47, 381–391. 10.1037/h0055392 13174710

[pri1814-bib-0012] Fonteyn, E. M. , Keus, S. H. , Verstappen, C. C. , Schols, L. , de Groot, I. J. , & Van de Warrenburg, B. P. (2014). The effectiveness of allied health care in patients with ataxia: A systematic review. Journal of Neurology, 261, 251–258. 10.1007/s00415-013-6910-6 23589192

[pri1814-bib-0013] Fonteyn, E. M. , Schmitz‐Hubsch, T. , Verstappen, C. C. , Baliko, L. , Bloem, B. R. , Boesch, S. , … van de Warrenburg, B. P. (2013). Prospective analysis of falls in dominant ataxias. European Neurology, 69, 53–57. 10.1159/000342907 23146840

[pri1814-bib-0014] Gibson‐Horn, C. (2008). Balance‐based torso‐weighting in a patient with ataxia and multiple sclerosis: A case report. Journal of Neurologic Physical Therapy, 32, 139–146. 10.1097/NPT.0b013e318185558f 18978670

[pri1814-bib-0015] Gorgas, A.‐M. , Widener, G. L. , Gibson‐Horn, C. , & Allen, D. D. (2015). Gait changes with balance‐based torso‐weighting in people with multiple sclerosis. Physiotherapy Research International, 20(1), 45–53. 10.1002/pri.1595 24930996 PMC4265579

[pri1814-bib-0016] Horak, F. , King, L. , & Mancini, M. (2015). Role of body‐worn movement monitor technology for balance and gait rehabilitation. Physical Therapy, 95, 461–470. 10.2522/ptj.20140253 25504484 PMC4348720

[pri1814-bib-0017] Hunt, C. M. , Widener, G. L. , & Allen, D. D. (2014). Variability in postural control with and without balance‐based torso‐weighting in people with multiple sclerosis and healthy controls. Physical Therapy, 94, 1489–1498. 10.2522/ptj.20130288 24903118 PMC4183891

[pri1814-bib-0018] Ilg, W. , Bastian, A. J. , Boesch, S. , Burciu, R. G. , Celnik, P. , Claasen, J. , … Timmann, D. (2014). Consensus paper: Management of degenerative cerebellar disorders. Cerebellum, 13, 248–268. 10.1007/s12311-013-0531-6 24222635 PMC4344126

[pri1814-bib-0019] Ilg, W. , Brotz, D. , Burkard, S. , Giese, M. A. , Schols, L. , & Synofzik, M. (2010). Long‐term effect of coordinative training in degenerative cerebellar disease. Movement Disorders, 25, 2239–2246. 10.1002/mds.23222 20737551

[pri1814-bib-0020] Ioffe, M. E. , Chernikova, L. A. , & Ustinova, K. I. (2007). Role of cerebellum in learning postural tasks. Cerebellum, 6(1), 87–94. 10.1080/14734220701216440 17366270

[pri1814-bib-0021] Lajoie, Y. , & Gallagher, S. P. (2004). Predicting falls within the elderly community: Comparison of postural sway, reaction time, the Berg balance scale and the Activities‐specific Balance Confidence (ABC) scale for comparing fallers and non‐fallers. Archives of Gerontology and Geriatrics, 38, 11–26. 10.1016/S0167-4943(03)00082-7 14599700

[pri1814-bib-0022] Leonardi, L. , Aceto, M. G. , Marcotulli, C. , Arcuria, G. , Serrao, M. , Pierelli, F. , … Casali, C. (2017). A wearable proprioceptive stabilizer for rehabilitation of limb and gait ataxia in hereditary cerebellar ataxias: A pilot open‐labeled study. Neurological Sciences, 38, 459–463. 10.1007/s10072-016-2800-x 28039539

[pri1814-bib-0023] Marquer, A. , Barbieri, G. , & Pérennou, D. (2014). The assessment and treatment of postural disorders in cerebellar ataxia: A systematic review. Annals of Physical and Rehabilitation Medicine, 57, 67–78. 10.1016/j.rehab.2014.01.002 24582474

[pri1814-bib-0024] Marsden, J. F. (2018). Cerebellar ataxia In Day B. L., & Lord S. R. (Eds.), Handbook of clinical neurology: Balance, gait, and falls (Vol. 159, 3rd series). Amsterdam, Netherlands: Elsevier.

[pri1814-bib-0025] Martino, G. , Ivanenko, Y. P. , Serrao, M. , Ranavolo, A. , d'Avella, A. , Draicchio, F. , … Lacquaniti, F. (2014). Locomotor patterns in cerebellar ataxia. Journal of Neurophysiology, 112, 2810–2821. 10.1152/jn.00275.2014 25185815

[pri1814-bib-0026] Mills, R. J. , Yap, L. , & Young, C. A. (2007). Treatment for ataxia in multiple sclerosis. Cochrane Database of Systematic Reviews. 10.1002/14651858.CD005029.pub2 17253537

[pri1814-bib-0027] Milne, S. C. , Corben, L. A. , Georgiou‐Karistianis, N. , Delatycki, M. B. , & Yiu, E. M. (2017). Rehabilitation for individuals with genetic degenerative ataxia: A systematic review. Neurorehabilitation and Neural Repair, 31(7), 609–622. 10.1177/1545968317712469 28595509

[pri1814-bib-0028] Miyai, I. , Ito, M. , Hattori, N. , Mihara, M. , Hatakenaka, M. , Yagura, H. , … Cerebellar Ataxia Rehabilitation Trialists Collaboration (2012). Cerebellar ataxia rehabilitation trial in degenerative cerebellar diseases. Neurorehabilitation and Neural Repair, 26, 515–522. 10.1177/1545968311425918 22140200

[pri1814-bib-0029] Morgan, M. H. (1975). Ataxia and weights. Physiotherapy, 61, 332–334. 1197409

[pri1814-bib-0030] Myers, A. M. , Fletcher, P. C. , Myers, A. H. , & Sherk, W. (1998). Discriminative and evaluative properties of the Activities‐specific Balance Confidence (ABC) Scale. Journal of Gerontology, 53A, M287–M294. 10.1093/gerona/53a.4.m287 18314568

[pri1814-bib-0031] Podsiadlo, D. , & Richardson, S. (1991). The timed "Up & Go": A test of basic functional mobility for frail elderly persons. Journal of the American Geriatrics Society, 39(2), 142–148. 10.1111/j.1532-5415.1991.tb01616.x 1991946

[pri1814-bib-0032] Portney, L. G. , & Watkins, M. P. (2009). Foundations of clinical research: Applications to practice (3rd ed.). Upper Saddle River, NJ: Pearson, Prentice Hall.

[pri1814-bib-0033] Rüb, U. , Brunt, E. R. , Seidel, K. , Gierga, K. , Mooy, C. M. , Kettner, M. , … Deller, T. (2008). Spinocerebellar ataxia type 7 (SCA7): Widespread brain damage in an adult‐onset patient with progressive visual impairments in comparison with an adult‐onset patient without visual impairments. Neuropathology and Applied Neurobiology, 34, 155–168. 10.1111/j.1365-2990.2007.00882.x 17971076

[pri1814-bib-0034] Salarian, A. , Horak, F. B. , Zampieri, C. , Carlson‐Kuhta, P. , Nutt, J. G. , & Aminian, K. (2010). ITUG, a sensitive and reliable measure of mobility. IEEE Trans Neural Syst Rehabil Eng, 18(3), 303–310. 10.1109/TNSRE.2010.2047606 20388604 PMC2922011

[pri1814-bib-0035] Sarva, H. , & Shanker, V. L. (2014). Treatment options in degenerative cerebellar ataxia: A systematic review. Movement Disorders Clinical Practice, 1(4), 291–298. 10.1002/mdc3.12057 30363941 PMC6183008

[pri1814-bib-0036] Schmitz‐Hubsch, T. , du Montcel, S. T. , Baliko, L. , Berciano, J. , Boesch, S. , Depondt, C. , … Fancellu, R. (2006). Scale for the assessment and rating of ataxia: Development of a new clinical scale. Neurology, 66, 1717–1720. 10.1212/01.wnl.0000219042.60538.92 16769946

[pri1814-bib-0037] Schmitz‐Hubsch, T. , Fimmers, R. , Rakowicz, M. , Rola, R. , Zdzienicka, E. , Fancellu, R. , … Klockgether, T. (2010). Responsiveness of different rating instruments in spinocerebellar ataxia patients. Neurology, 74, 678–684. 10.1212/WNL.0b013e3181d1a6c9 20177122

[pri1814-bib-0038] Schniepp, R. , Wuehr, M. , Schlick, C. , Huth, S. , Pradhan, C. , Dieterich, M. , … Jahn, K. (2014). Increased gait variability is associated with the history of falls in patients with cerebellar ataxia. Journal of Neurology, 261, 213–223. 10.1007/s00415-013-7189-3 24263407

[pri1814-bib-0039] Spain, R. I. , George, S. R. J. , Salarian, A. , Mancini, M. , Wagner, J. M. , Horak, F. , & Bourdette, D. (2012). Body‐worn motion sensors detect balance and gait deficits in people with multiple sclerosis who have normal walking speed. Gait & Posture, 35, 573–578. 10.1016/j.gaitpost.2011.11.026 22277368 PMC3614340

[pri1814-bib-0040] Stephen, C. D. , Brizzi, K. T. , Bouffard, M. A. , Gomery, P. , Sullivan, S. L. , Mello, J. , … Schmahmann, J. D. (2019). The comprehensive management of cerebellar ataxia in adults. Current Treatment Options in Neurology, 21, 9.1–9.17. 30788613 10.1007/s11940-019-0549-2

[pri1814-bib-0041] Stolze, H. , Klebe, S. , Petersen, G. , Raethjen, J. , Wenzelburger, R. , Witt, K. , & Deuschl, G. (2002). Typical features of cerebellar ataxic gait. Journal of Neurology, Neurosurgery and Psychiatry, 73(3), 310–312. 10.1136/jnnp.73.3.310 PMC1738052 12185166

[pri1814-bib-0042] Thabane, L. , Ma, J. , Chu, R. , Cheng, J. , Ismaila, A. , Rios, L. P. , … Goldsmith, C. H. (2010). A tutorial on pilot studies: The what, why and how. BMC Medical Research Methodology, 10, 1 10.1186/1471-2288-10-1 20053272 PMC2824145

[pri1814-bib-0043] Van de Warrenburg, B. , Steijns, M. A. , Munneke, M. , Kremer, B. , & Bloem, B. R. (2005). Falls in degenerative cerebellar ataxias. Movement Disorders, 20, 497–508. 10.1002/mds.20375 15645525

[pri1814-bib-0044] Whitepaper (2015). Mobility Lab by APDM, Inc , from http://www.apdm.com/wp-content/uploads/2015/08/Whitepaper1.pdf

[pri1814-bib-0045] Widener, G. L. , Allen, D. D. , & Gibson‐Horn, C. (2009). Randomized clinical trial of balance‐based torso weighting for improving upright mobility in people with multiple sclerosis. Neurorehabilitation and Neural Repair, 23(8), 784–791. 10.1177/1545968309336146 19470807

[pri1814-bib-0046] Wrisley, D. M. , & Whitney, S. L. (2004). The effect of foot position on the modified clinical test of sensory interaction and balance. Archives of Physical Medicine & Rehabilitation, 85, 335–338. 10.1016/j.apmr.2003.03.005 14966723

[pri1814-bib-0047] Wuehr, M. , Schniepp, R. , Ilmberger, J. , Brandt, T. , & Jahn, K. (2013). Speed‐dependent temporospatial gait variability and long‐range correlations in cerebellar ataxia. Gait & Posture, 37, 214–218. 10.1016/j.gaitpost.2012.07.003 22840892

